# Global multisensory reorganization after vestibular brain stem stroke

**DOI:** 10.1002/acn3.51161

**Published:** 2020-08-28

**Authors:** Julian Conrad, Maximilian Habs, Rainer Boegle, Matthias Ertl, Valerie Kirsch, Iskra Stefanova‐Brostek, Ozan Eren, Sandra Becker‐Bense, Thomas Stephan, Frank Wollenweber, Marco Duering, Peter zu Eulenburg, Marianne Dieterich

**Affiliations:** ^1^ Department of Neurology University Hospital LMU Munich Munich Germany; ^2^ German Center for Vertigo and Balance Disorders (DSGZ) University Hospital LMU Munich Munich Germany; ^3^ Graduate School of Systemic Neurosciences – GSN‐LMU LMU Munich Munich Germany; ^4^ Department of Psychology University of Bern Bern Switzerland; ^5^ Department of Neurology Muenchen Klinik Harlaching Germany; ^6^ Institute for Stroke and Dementia Research (ISD) University Hospital LMU Munich Munich Germany; ^7^ Department of Neurology Helios Dr. Horst Schmidt Kliniken Wiesbaden Germany; ^8^ Institute for Neuroradiology University Hospital LMU Munich Munich Germany; ^9^ Munich Cluster for Systems Neurology (SyNergy) Munich Germany

## Abstract

**Objective:**

Patients with acute central vestibular syndrome suffer from vertigo, spontaneous nystagmus, postural instability with lateral falls, and tilts of visual vertical. Usually, these symptoms compensate within months. The mechanisms of compensation in vestibular infarcts are yet unclear. This study focused on structural changes in gray and white matter volume that accompany clinical compensation.

**Methods:**

We studied patients with acute unilateral brain stem infarcts prospectively over 6 months. Structural changes were compared between the acute phase and follow‐up with a group of healthy controls using voxel‐based morphometry.

**Results:**

Restitution of vestibular function following brain stem infarcts was accompanied by downstream structural changes in multisensory cortical areas. The changes depended on the location of the infarct along the vestibular pathways in patients with pathological tilts of the SVV and on the quality of the vestibular percept (rotatory vs graviceptive) in patients with pontomedullary infarcts. Patients with pontomedullary infarcts with vertigo or spontaneous nystagmus showed volumetric increases in vestibular parietal opercular multisensory and (retro‐) insular areas with right‐sided preference. Compensation of graviceptive deficits was accompanied by adaptive changes in multiple multisensory vestibular areas in both hemispheres in lower brain stem infarcts and by additional changes in the motor system in upper brain stem infarcts.

**Interpretation:**

This study demonstrates multisensory neuroplasticity in both hemispheres along with the clinical compensation of vestibular deficits following unilateral brain stem infarcts. The data further solidify the concept of a right‐hemispheric specialization for core vestibular processing. The identification of cortical structures involved in central compensation could serve as a platform to launch novel rehabilitative treatments such as transcranial stimulations.

## Introduction

Acute central vestibular syndrome manifests with rotational vertigo, spontaneous nystagmus (SPN), tilts of the subjective visual vertical (SVV), and postural instability with lateral falls. Vestibular symptoms recover within weeks, a process known as central vestibular compensation.^1^, ^2^, ^3^ Central compensation of a unilateral *peripheral* loss of vestibular function is based on multiple processes that occur in distributed neuronal networks at different locations and at different times.^4^, ^5^, ^6^, ^7^ Unique features within the vestibular system have to be taken into account: In contrast to other sensory systems, vestibular signals are integrated early with other sensory input in the lower brain stem, and thus cortical vestibular areas are always multisensory as they respond to several sensory stimuli.^8^, ^9^, ^10^ The structural basis of compensation is the bilaterally organized vestibular system. The sensory signals are conveyed from the vestibular end organs to the vestibular nuclei in the pontomedullary brain stem and via several bilateral pathways with multiple crossings to the thalamus. From there, they reach the multisensory integration centers of the temporoparietal cortex.^11^, ^12^, ^13^, ^14^, ^15^ The posterior insula with the parietal opercular cortex (OP2), the posterior insular and retroinsular cortex were reliably identified as the core regions of the multisensory vestibular network in humans.^14^, ^16^, ^17^, ^18^, ^19^ These regions show a right‐hemispheric preponderance for vestibular signal processing in right‐handed humans.^18^

Some structural compensatory changes have been demonstrated for peripheral vestibular lesions.^20^, ^21^, ^22^ Central compensation in *central* vestibular lesions has only been investigated in a few studies using PET and functional MRI.^23^, ^24^, ^25^, ^26^

The current study used voxel‐based morphometry (VBM) to evaluate changes in gray matter volume (GMV) and white matter volume (WMV) over time in 24 patients with acute unilateral brain stem infarcts presenting with vestibular or ocular motor deficits, compared to the baseline (acute phase).^27^ A group of healthy age‐ and gender‐matched participants (HC) served as a control group.

There should be no differences in GMV and WMV between the HC and the patients in the acute phase, but changes were expected after 6 months. The following questions were addressed: (i) Are the structural changes dependent on the location of the infarct, that is, pontomedullary vs pontomesencephalic lesions? (ii) Which sensorimotor areas are particularly involved, those of the multisensory vestibular network only or also areas belonging to the visual and somatosensory system? This is important to evaluate the contribution of substitution to central compensation. (iii) Do the compensatory structural changes reflect the vestibular dominance of the right hemisphere and the upper brain stem as identified by functional imaging and functional connectivity MRI? Are the compensatory changes in the brain stem and cerebellum symmetric or asymmetric?

## Materials and Methods

### Standard protocols and procedures

The study was performed in accordance with the 1964 Declaration of Helsinki (latest applicable revision Fortaleza 2013) and approved by the institutional review board of LMU Munich, Germany (no.094‐10). All patients gave informed written consent to participate in the study.

### Patients

We included 24 patients with ischemic brain stem infarcts who presented to our tertiary referral center (University Hospital, LMU Munich, Germany) between 2012 and 2019. Inclusion criteria were as follows: Imaging confirmed unilateral brain stem infarct, ability to complete the detailed vestibular and ocular motor examination, completion of follow‐up imaging, vestibular and ocular motor examination after 6 months. Exclusion criteria were as follows: Absence of an ischemic lesion on diffusion MRI, clinically confirmed peripheral vestibular deficit, bilateral or multifocal infarcts, prior stroke, tumor, cerebral hemorrhage, vascular malformation, edema (i.e., compression of CSF space, shift of midline structures), severe white matter hyperintensities (WMH, Fazekas grade> 1 for periventricular WMH and deep WMH), and if patients were unable to complete the neurological and neuro‐ophthalmological examination due to cognitive impairment or impaired vigilance.^28^

All patients received a complete clinical and radiological work‐up in the acute (M0) and chronic (6‐month follow‐up, M6) stage. We had to exclude some patients due to loss to follow‐up (*n* = 8); insufficient clinical data (*n* = 9); missing structural imaging/poor imaging data quality (*n* = 4).

### Controls

We also examined a group of 38 age‐ and gender‐matched right‐handed healthy controls with an identical imaging protocol. The control population had no prior history of peripheral or central vestibular disorders.

### Clinical examination

All patients received a thorough clinical and neuro‐orthoptic examination, including measurements of the SVV.^11^ In a subset of patients (*n* = 3 acute, *n* = 17 chronic) the SVV was determined using the bucket test (Table 1).^29^

**Table 1:** Prevalence of clinical deficits.

	Pontomedullary lesions				Pontomesencephalic lesions			
	M0		M6		M0		M6	
SVV mean (SD; °)	+/− 4.46 (+/−3.83)		+/− 2.19 (+/− 1.5)		+/− 4.19° (+/−3.12)		+/− 1.92 (+/−1.06)	
Clinical Test	*n*	%	*n*	%	*n*	%	*n*	%
Vestibular								
Pathological SVV score	9/15	60.0	1/15	6.7	7/9	77.8	1/9	11.1
Skew deviation	6/15	40.0	0/15	0	2/9	22.2	2/9	0
Ocular torsion	6/15	40.0	0/15	0	2/8	25.0	0/8	0
Head tilt	3/15	20.0	0/15	0	1/9	11.1	1/9	0
Spontaneous nystagmus (SPN)	9/15	66.6	0/15	0	3/9	33.3	0/9	0
Pathological VOR	1/15	6.7	1/15	6.7	0/9	0.0	0/9	0
Lateropulsion	2/15	13.3	1/15	6.7	1/9	11.1	0/9	0
Ocular motor								
Gaze‐evoked nystagmus	7/15	46.7	1/15	6.7	4/9	44.4	0/9	0
Saccadic smooth pursuit	13/15	86.7	3/15	26.7	7/9	77.8	6/9	66.7
Dysmetria of saccades	8/15	53.3	0/15	0	1/9	11.1	3/9	33.3
Saccade palsy	4/15	26.7	0/15	0	4/9	44.4	0/9	0
Gaze palsy	1/15	6.7	0/15	0	2/9	22.2	1/9	11.1
Path. optokinetic reflex	2/15	13.3	0/15	0	4/9	44.4	0/9	0
Path. fixation suppression of VOR	3/15	20.0	0/15	0	2/9	22.2	1/9	11.1
Ocular motor cranial nerve palsy^1^	2/15	13.3	0/15	0	1/9	11.1	1/9	11.1
INO	1/15	6.7	0/15	0	4/9	44.4	0/9	0
Vestibular/ ocular motor subjective								
Rotational vertigo	9/15	60.0	0/15	0	4/9	44.4	0/9	0
Double vision	3/15	20.0	1/15	6.7	4/9	44.4	0/9	0
Tendency to fall	8/15	53.3	2/15	13.3	3/9	33.3	0/9	0
Higher order multisensory								
Spatial neglect	0/15	0	0/15	0	0/9	0	0/9	0
Pushing behavior	0/15	0	0/15	0	0/9	0	0/9	0
Non‐vestibular/ ocular motor symptoms								
Paresis (hemiparesis, limb)	5/15	33.3	1/15	6.7	4/9	44.4	1/9	11.1
Hypesthesia	11/15	73.3	3/15	26.7	3/9	33.3	0/9	0
Limb ataxia	5/15	33.3	0/15	0	3/9	33.3	1/9	11.1
Dysarthria	9/15	60.0	0/15	0	4/9	44.4	0/9	0
Horner syndrome	3/15	20.0	1/15	6.7	0/9	0	0/9	0

^1^pontomedullary infarcts: 2/15 VI palsy, pontomesencephalic infarcts: 1/9 III palsy.

### Imaging

All patients underwent high‐resolution structural MRI on a clinical 3T MRI scanner (13 patients: GE Signa Excite HD, Milwaukee, WI, USA, T1FSPGR, 1 mm^3^ isotropic, 176 slices, TR 6.63 ms, TE 3.15 ms; 11 patients: T1MPRAGE, 1 mm^3^ isotropic, 192 slices, TR 2500 ms, TE 4.37 ms, Magnetom Verio or Magnetom Skyra, Siemens Healthcare, Erlangen, Germany, part of the DEDEMAS study).^30^ All patients had their longitudinal MRIs on the same scanner. Correspondingly, the group of healthy controls was examined on the MRI machines mentioned above (GE *n* = 23, Siemens *n* = 17).

Data quality estimation, preprocessing, and analysis were performed using the CAT12 toolbox, version 1450 (http://www.neuro.uni‐jena.de/cat) within Statistical Parametric Mapping SPM12, version 7487 (https://www.fil.ion.ucl.ac.uk/spm/; Wellcome Department of Cognitive Neurology), using Matlab R2017b (Mathworks) after standard preprocessing including an 8‐mm Gaussian smoothing kernel. The modulated GM and WM images were used for the volumetric analysis. Sample homogeneity analysis revealed an excellent correlation within the sample. The infarcts were delineated on diffusion‐weighted imaging (DWI, 2 mm, TR 8000 ms, TE 88 ms, 30 slices) using MRICRON (https://people.cas.sc.edu/rorden/mricron/index.html). Lesion maps were then normalized into MNI space using the *Clinical Toolbox* in SPM (https://www.fil.ion.ucl.ac.uk/spm).^31^

### Statistical analysis

Data of time point M0, M6, and of the HC (one time point) were included in a 2x2 ANOVA approach (mixed model) with group (HC, pontomedullary, pontomesencephalic) and time point (M0, M6, controls M0 only) as factors. Total intracranial volume (TIV), age, and time to follow‐up were used as covariates of no interest.

For the group with pontomedullary infarcts, analyses were conducted for those with spontaneous nystagmus (SPN, *n* = 9/15), deviation of the SVV (*n* = 9/15), and for patients with rotational vertigo (*n* = 9/15). We chose a dichotomous categorization for the analysis of SVV tilts (pathological/ not pathological). In the group of patients with pontomesencephalic infarcts only the patients with pathological tilt of the SVV (*n* = 7/9) were analyzed as a group because nystagmus and rotational vertigo were present in less than 50% of the cases and did not allow for further subcategorization. T contrasts were estimated to detect differences between the groups. The results were further analyzed using nonparametric permutation testing (*threshold‐free cluster enhancement, TFCE*) as implemented in the CAT12 toolbox calculating 5000 permutations.^32^ *TFCE* is threshold free, sensitive for high focal as well as widely distributed low effects (cluster enhancement), nonparametric, and does not interfere with focal changes in smoothness.^33^ All results were corrected for multiple comparisons on the cluster level using *family‐wise error (FWE)* correction; p < 0.05. Changes in GMV and WMV were projected onto a MNI152 template brain using *MRICROGL* (https://www.mccauslandcenter.sc.edu/mricrogl/).

### Interhemispheric differences of volumetric changes in homologous brain regions

To account for interhemispheric differences of signal changes, we compared cluster size (in voxels) and peak signal intensity (T score) on a whole brain level (all significant clusters in the left vs right hemisphere). In the second step, we compared only those homologous brain areas that process vestibular information in both hemispheres (cerebellum, brain stem, thalamus, insular and parietal opercular cortex (this includes areas Ig1, Ig2, retroinsular cortex, OP), cingulate cortex, intraparietal sulcus (IPS)/superior parietal lobule (SPL); Wilcoxon signed‐rank test, *P* < 0.05).

### Data availability statement

The dataset is not publicly available due to European Privacy laws and lack of consent for publication by the patients.

## Results

### Sociodemographic

There was no significant difference in age or handedness between the patient and control groups: median 68 years (range: 28–86 years) in the patient group vs 68 years (51–79 years) in the control group; 100% right‐handed in the patient group vs. 97.3% (HC).

### Clinical

**Figure 1:**
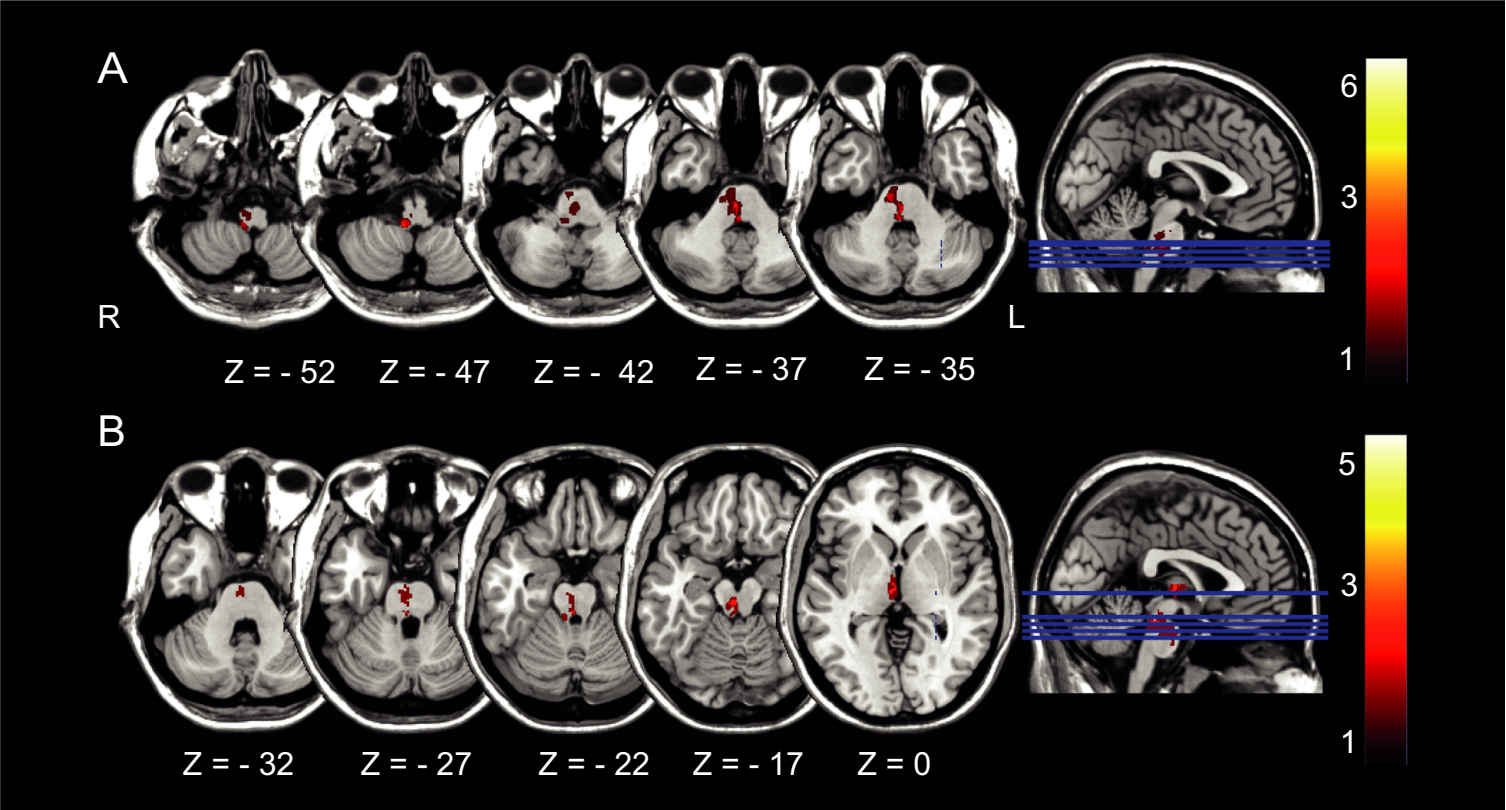
Distribution of (A) pontomedullary infarcts and (B) pontomesencephalic infarcts. Color bar indicates the converging number of patients affected at a specific voxel location which demonstrates heterogeneity in terms of lesion location but homogeneity in clinical presentation. All lesions were flipped to the right to create this overlap image.

Infarcts were termed as pontomedullary using the final MRI lesions (pontomedullary infarcts extended to the vestibular nuclei complex). Fifteen patients had pontomedullary infarcts without evidence of a concurrent cerebellar lesion and nine patients had pontomesencephalic infarcts. Patients with affection of the vestibular pathways to the ocular motor centers in the rostral midbrain and collateral paramedian thalamic lesions were included in the group of pontomesencephalic infarcts (*n* = 2). One pontomesencephalic and one pontomedullary infarct extended into the territory of the other group. In both cases > 80% of the infarct lay in the territory to which it was assigned. Clinical deficits were not significantly different between the groups and were compensated at M6 (no SPN and no rotational vertigo, one borderline pathological but significantly improved SVV score in each group of pontomedullary and pontomesencephalic infarcts without clinical symptoms, Table 1). Unilateral infarcts were distributed similarly on both sides in the group of pontomedullary infarcts (*n* = 8 left, *n* = 7 right). The majority of the nine pontomesencephalic infarcts were left sided (*n* = 6). There was no size difference between left‐ and right‐sided infarcts (pontomedullary: *Mann‐Whitney U‐Test* *P* = 0.779; pontomesencephalic: *P* = 0.30, Fig. 1).

### Pontomedullary infarcts with spontaneous nystagmus (SPN) and rotational vertigo

#### GMV

**Figure 2:**
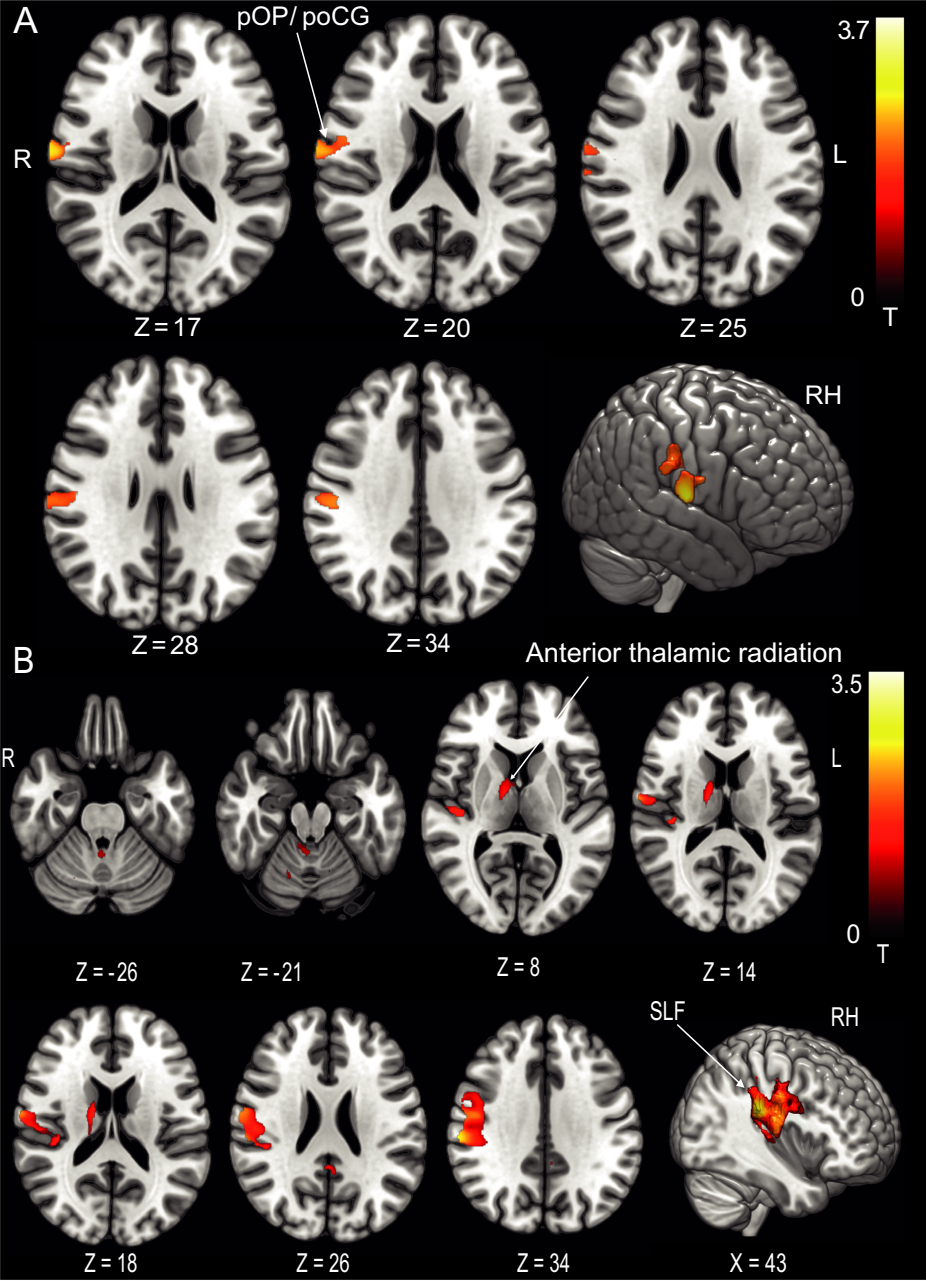
Pontomedullary infarcts. (A) GMV and (B) WMV increases at follow‐up after 6 months in the group of patients with spontaneous nystagmus compared to the healthy control group (*n* = 9). GMV increases were located in the parietal opercular cortex/postcentral gyrus of the right hemisphere only, an additional WMV increase was evident in the anterior thalamic radiation in the right hemisphere. This further solidifies the concept of a dominance of the vestibular system in humans with a lateralization to the right in right handers (*P* < 0.05, FWE corrected). OP parietal opercular, poCG postcentral gyrus, SLF superior longitudinal fascicle.

**Figure 3:**
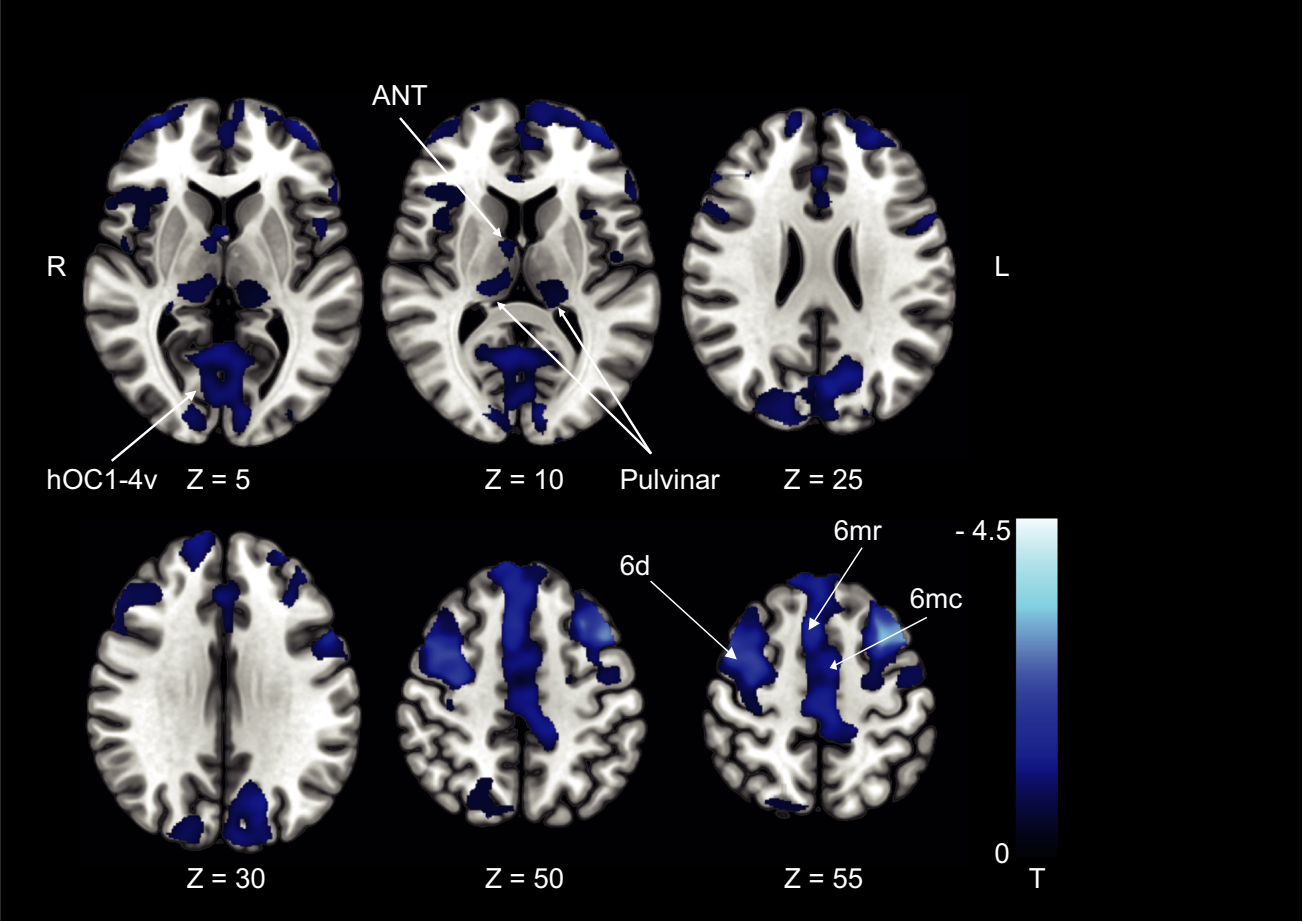
Pontomedullary infarcts. GMV decreases at follow‐up after 6 months in the group of patients with spontaneous nystagmus compared to the control group (*n* = 9). GMV decreases were located in premotor cortex (6d, 6mc, 6mr) and ventral visual streams (hOC1‐4v; thresholded at *P* < 0.001, FWE corrected for visualization). ANT, anterior thalamic nuclei; MD, mediodorsal nucleus; hOC, human occipital cortex.

The patterns of changes in GMV and WMV for patients with SPN were similar to those with rotational vertigo. While there were no differences between patients in the acute phase (M0) and the HC, increases in GMV at M6 compared to the HC were found in the parietal opercular cortex and postcentral gyrus of the right hemisphere only. GMV decreases were located bilaterally in the cerebellar hemispheres (crus I, lobule VI, VIIa/b) and cerebellar vermis (lobules X, IX, VIIIa,b), the pulvinar, the anterior thalamic nuclei (ANT) extending to the mediodorsal nucleus (MD) bilaterally and in the premotor cortex (cytoarchitectonic areas 6d, 6mc, 6mr). Additional decreases were found along the ventral visual stream bilaterally (hOC 1‐4v, area FG3 extending to the hippocampus; Fig. 2A, Fig. 3, for results of GMV in patients with rotational vertigo, see summary Fig. 6).

#### WMV

WMV increases were located, correspondingly, in the WM around the parietal opercular cortex (cytoarchitectonic areas Ig1, Ig2, TE1, OP1, OP2, this area also includes the retroinsular cortex which is not part of the Anatomy Toolbox) and postcentral gyrus of the right hemisphere. Small clusters were found in the cerebellar hemispheres and cerebellar vermis, cingulate cortex and anterior thalamic radiation of the right hemisphere (Fig. 2B).

### Pontomedullary infarcts with deviations of the SVV

#### GMV

**Figure 4:**
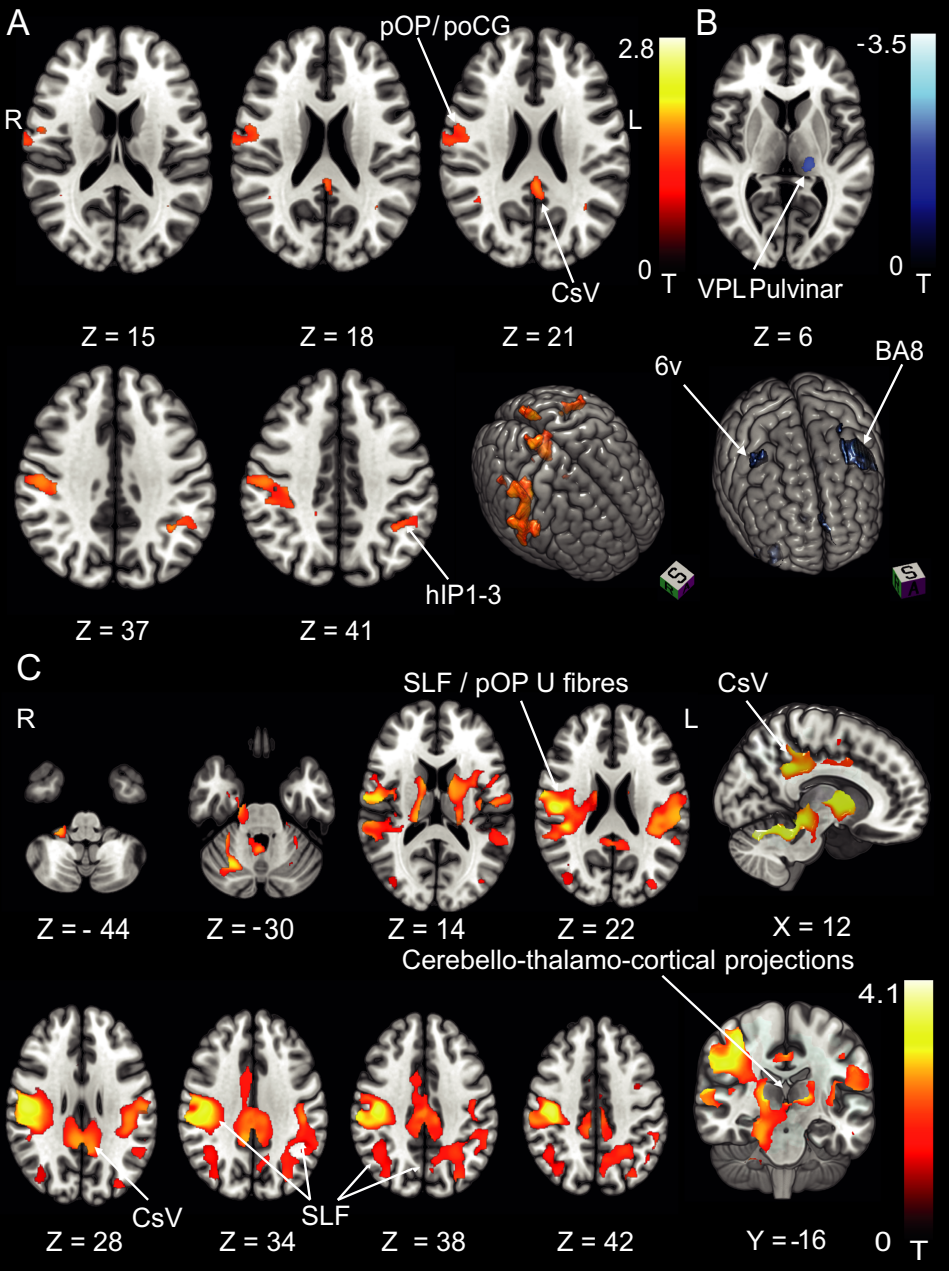
Pontomedullary infarcts. (A) GMV increases (B) GMV decreases, and (C) WMV increases at follow‐up after 6 months in the group of patients with deviation of the SVV compared to the control group (*n* = 9). GMV increases were located in the parietal opercular cortex and along the postcentral gyrus extending to the IPS and SPL in the right hemisphere and the IPS in the left hemisphere. GMV decreases were located in premotor area 6v and FEF (BA8) and the left ventral posterior lateral nucleus of the thalamus and pulvinar. WMV increases were located in the posterior sections of the superior longitudinal fascicle (with stronger response in the right hemisphere, around the cingulate visual area and cerebello‐thalamocortical WM projections. The amount of structural changes in both hemispheres represents the importance of multisensory adaptation and substitution for graviceptive processing compared to the changes within the right hemisphere only for the processing of semicircular canal‐derived vestibular signals (rotational vertigo and nystagmus). A, C *P* < 0.05, FWE corrected, B thresholded at *P* < 0.001, FWE corrected for visualization). pOP, parietal opercular; poCG, postcentral gyrus; hIP, human intraparietal sulcus; pCG VA, posterior cingulate cortex visual area; SLF, superior longitudinal fascicle; VPL, ventral posterior lateral nucleus.

In the chronic stage, there was an increase in GMV around the parietal opercular cortex extending along the postcentral gyrus of the right hemisphere compared to the HC. Additional clusters were located in the superior parietal lobule (5L, M in the right and 7A in the left hemisphere), around the left > right anterior intraparietal sulcus (IPS) and posterior cingulate cortex bilaterally (Fig. 4A). GM decreases were found in premotor area 6v adjacent to the frontal eye fields (FEF, BA8) bilaterally and the left ventral posterior lateral thalamic nucleus (VPL, Fig. 4B).

#### WMV

WMV increases were located within the parietal opercular cortex (around cytoarchitectonic areas TE1, Ig1, Ig2, OP2, OP3 includes the retroinsular cortex, see above) and adjacent to the postcentral gyrus in both hemispheres with larger clusters in the right hemisphere. Clusters extended to the posterior parietal cortex in both hemispheres. Subcortical WMV increases were found from the flocculus and cerebellar hemispheres via the superior cerebellar peduncle (SCP) and the cerebello‐thalamocortical tract to the parietal cortex of both hemispheres. While increases were rather symmetric in the cerebellum, there was a preponderance of right‐sided increases in the upper brain stem and parietal opercular areas (Fig. 4C).

### Pontomesencephalic infarcts with deviations of the SVV

#### GMV

**Figure 5:**
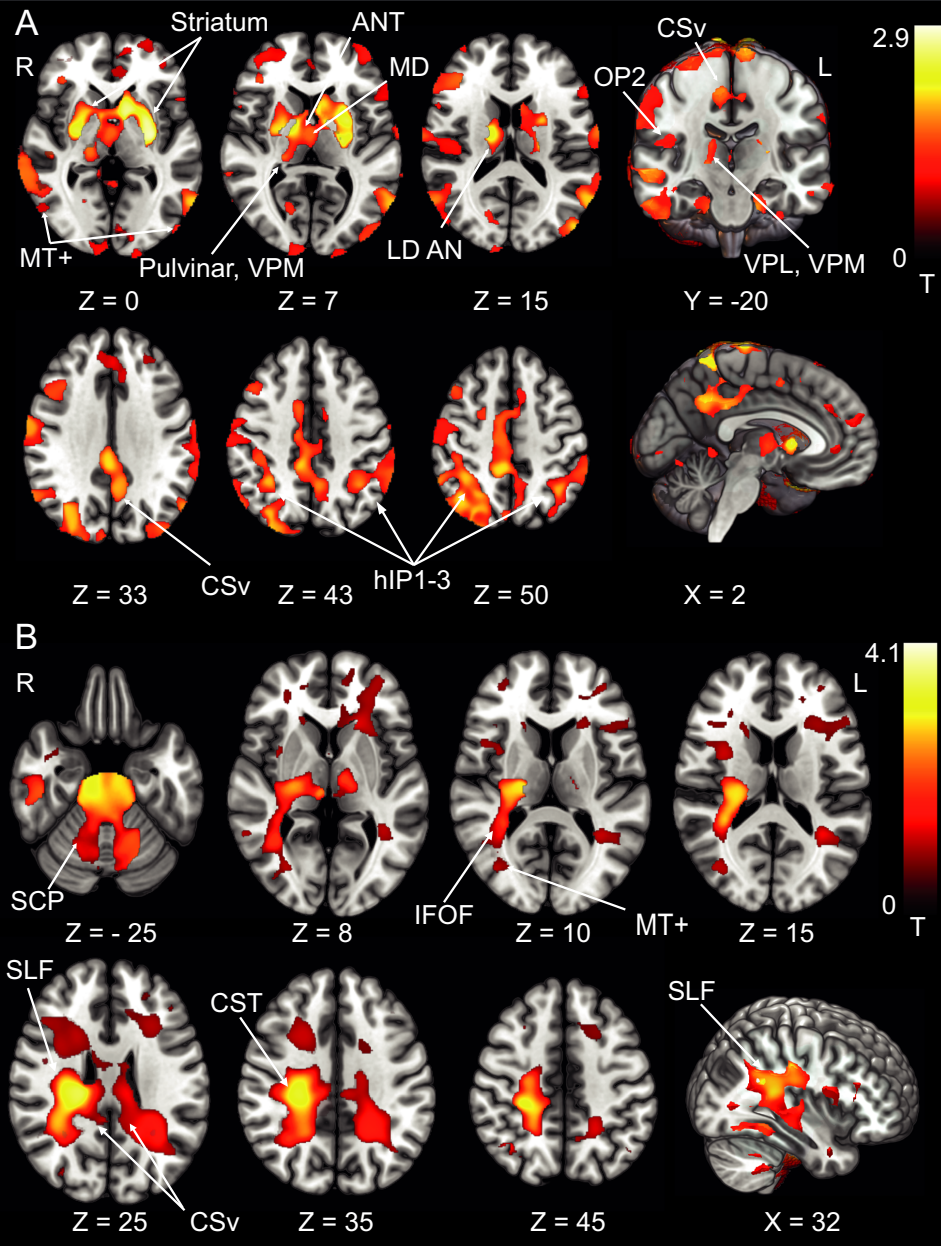
Response pattern in pontomesencephalic infarcts. (A) GMV increases and (B) WMV increases at follow‐up after 6 months in the group of patients with deviations of the SVV compared to the control group (*n* = 7). In contrast to pontomedullary lesions, pontomesencephalic lesions with tilts of the SVV showed structural adaptive changes in all areas of the cortical vestibular circuitry of both hemispheres as well as the striatum which might represent the motor integration of vestibular output function (postural head/trunk control; A thresholded at *P* < 0.05, FWE corrected, B at *P* < 0.001, FWE corrected for visualization). ANT, anterior thalamic nuclei; LD, laterodorsal nucleus; MD, mediodorsal nucleus; VPL, ventral posterior lateral nucleus; VPM, ventral posterior medial nucleus; OP2, parietal opercular cortex; hIP, human intraparietal sulcus; CSv, cingulate sulcus visual area. MT + motion‐sensitive middle temporal area; SCP, superior cerebellar peduncle; IFOF, inferior fronto‐occipital fascicle; CST, corticospinal tract; SLF, superior longitudinal fascicle.

At M6 compared to the HC, GMV increases were located in the right cerebellar hemisphere (VIIa,b, VIIIa, Crus I, II), the striatum, and paramedian and posterolateral thalamus of the right hemisphere. Cortical GMV increases involved the parietal opercular cortex (RH: OP1, OP2, LH: OP1, OP4) in both hemispheres extending to the IPS bilaterally, the posterior cingulate cortex, and motion‐sensitive middle temporal areas (MT+) in both hemispheres (Fig. 5A).

#### WMV

WMV increases were located in both cerebellar hemispheres and the brain stem, the SLF adjacent to cytoarchitectonic area OP2, Ig1 and Ig2, TE1 (includes the retroinsular cortex, see above) of the right hemisphere, and the posterior cingulate cortex (CSv) as well as the corticospinal tract (CST) bilaterally. Additional clusters were found in the WM beneath the IFG and the MT + region bilaterally (see Fig. 5B for a detailed depiction).

### Interhemispheric differences in cluster size and peak signal increases

Volumetric increases were larger in the right compared to the left hemisphere (*P* = 0.012, *Wilcoxon signed‐rank test*) for pontomedullary and pontomesencephalic infarcts (Table 2A) on a whole brain level. However, when considering the known central vestibular sites alone, only the clusters in the parietal opercular cortex showed this effect (*P* = 0.012). When using the peak T score intensity, only the difference between right and left parietal opercular cortex was significant (Table 2B).

**Table 2:** (A) Interhemispheric differences in infarct response cluster size. (B) Interhemispheric differences in infarct response peak intensity (T score). Wilcoxon signed‐rank, test results at the bottom of each partition (*P* < 0.05).

A. Pontomedullary and pontomesencephalic infarcts														
	All Clusters		Parietal Opercular		Cerebellar		Brainstem		Cingulate		Thalamus		IPS/ SPL	
	RH	LH	RH	LH	RH	LH	RH	LH	RH	LH	RH	LH	RH	LH
PontoMed all GM	34365	23510	17213	0	0	3308	0	0	6545	6545	0	0	4704	16331
PontoMed all WM	53813	2597	26640	0	6680	3080	2400	522	2102	2102	0	0	1280	592
PontoMed SPN GM	4137	0	4137	0	0	0	0	0	0	0	0	0	0	0
PontoMed SPN WM	20624	142	16421	0	1252	142	0	0	709	709	2242	0	0	0
PontoMed SVV GM	15980	7456	11508	0	0	0	0	0	0	2887	0	0	2563	3608
PontoMed SVV WM	132837	71300	50836	28312	15864	5956	4660	544	11660	9676	15240	1428	6352	15904
PontoMes SVV GM	187056	148700	5512	5132	11416	0	0	0	2708	4700	5632	1680	39240	12428
PontoMes SVV WM	127344	62496	70628	40156	14920	14900	na	na	0	0	na	na	70628	40156
Z	−2.521		−2.521		−1.572		−1.342		−1.069		−1.604		−0.314	
*P* (two‐sided)	**0.012**		**0.012**		0.116		0.180		0.285		0.109		0.753	
Pontomedullary infarcts only														
Z	−2.201		−2.201		−1.095		−1.342		−0.447		−1.342		0.000	
*P* (two‐sided)	**0.028**		**0.028**		0.273		0.180		0.655		0.180		1.000	

B. Pontomedullary and pontomesencephalic infarcts														
	All Clusters		Parietal Opercular		Cerebellar		Brainstem		Cingulate		Thalamus		IPS/ SPL	
	RH	LH	RH	LH	RH	LH	RH	LH	RH	LH	RH	LH	RH	LH
PontoMed all GM	2.8	2.8	2.4	1.6	1.8	1.8	0	0	2.2	2.8	0.6	0.3	2.7	2.3
PontoMed all WM	3.5	3.1	3.5	2.5	3.3	2.6	3.2	1.7	2.9	2.9	2.2	2.9	2.8	3.1
PontoMed SPN GM	3.7	0	3.7	0	0	0	0	0	2.1	3.1	0	0	0	0
PontoMed SPN WM	3.8	2.9	3.8	0	0	0	0	0	2.9	2.9	2.9	0	0	0
PontoMed SVV GM	2.7	3.1	2.6	0	0	0	0	0	2.7	2.7	0	0	2.6	3.2
PontoMed SVV WM	3.6	3.2	3.4	2.9	3.6	3.2	2.3	0	3.1	3.1	4.0	3.6	3.3	3.3
PontoMes SVV GM	3.5	2.4	2.6	2.2	3.5	1.7	0	0	2.4	2.4	2.0	1.0	2.1	2.1
PontoMes SVV WM	4.0	2.6	4.0	3.0	3.3	2.7	2.2	3.1	0	0	2.7	2.1	2.7	2.4
Z	−1.86		‐2.52		−1.83		−1.07		0		−1.36		−0.184	
*P* (two‐sided)	0.063		**0.012**		0.068		0.285		1		0.173		0.854	
Pontomedullary infarcts only														
Z	−1.22		−2.20		−1.34		−1.34		0		−0.73		−0.535	
*P* (two‐sided)	0.223		**0.028**		0.180		0.180		1		0.465		0.593	

### Discussion

The main findings of the study are as follows: (i) Downstream structural volumetric changes following brain stem infarcts take place in multiple sensory and motor regions in both hemispheres. These changes accompany clinical compensation. This was evident in patients with pontomedullary and pontomesencephalic infarcts alike. (ii) The volume increases in multisensory vestibular cortical areas showed a right‐hemispheric preference. (iii) Compensation of spontaneous nystagmus and rotational vertigo in pontomedullary brain stem infarcts was accompanied by GMV and WMV increases in the core cortical vestibular areas and ventral parts of the postcentral gyrus in the right hemisphere only. (iv) Compensation of graviceptive dysfunction (i.e., SVV tilts) led to large supplementary GMV and WMV increases bilaterally in the parietal and postcentral (somatosensory) cortex and along the white matter tracts that connect the parietal opercular cortex and intraparietal sulcus with the premotor cortex. (v) Volumetric increases were located primarily in multisensory areas in pontomedullary infarcts. In pontomesencephalic infarcts, additional increases were found in motor and middle temporal areas. (vi) Volume decreases after pontomedullary brain stem infarcts involved the visual and motor systems.

### Central compensation of vestibular syndromes

A few studies have already demonstrated cortical changes following chronic peripheral vestibular lesions.^21^, ^22^ Studies on central compensation of *central* vestibular lesions are scarce. Bense and colleagues found signal decreases in visual cortex in pontomedullary infarcts and decreases in the premotor cortex in both pontomedullary and pontomesencephalic infarcts with functional imaging.^25^, ^26^ However, structural plasticity, that is, an increase of GMV and WMV in the cortical multisensory vestibular areas following unilateral brain stem infarcts has not been demonstrated before. Apart from the inherent differences in these two methods, we used a thorough state‐of‐the‐art preprocessing and analysis algorithm that allows subtle changes in GMV to be detected and is corrected for type 1 errors (*TFCE* with *FWE* correction for multiple comparisons) which could additionally explain the differences between ours and the former studies.

Significantly, we found a right‐hemispheric dominance for GMV and WMV increases in the parietal opercular multisensory vestibular and somatosensory areas for patients with pontomedullary infarcts presenting with the unique vestibular symptoms of spontaneous nystagmus and rotatory vertigo. This is in line with the known lateralization of the vestibular system after caloric irrigation with a dominance of the right hemisphere in right handers.^18^, ^34^

**Figure 6:**
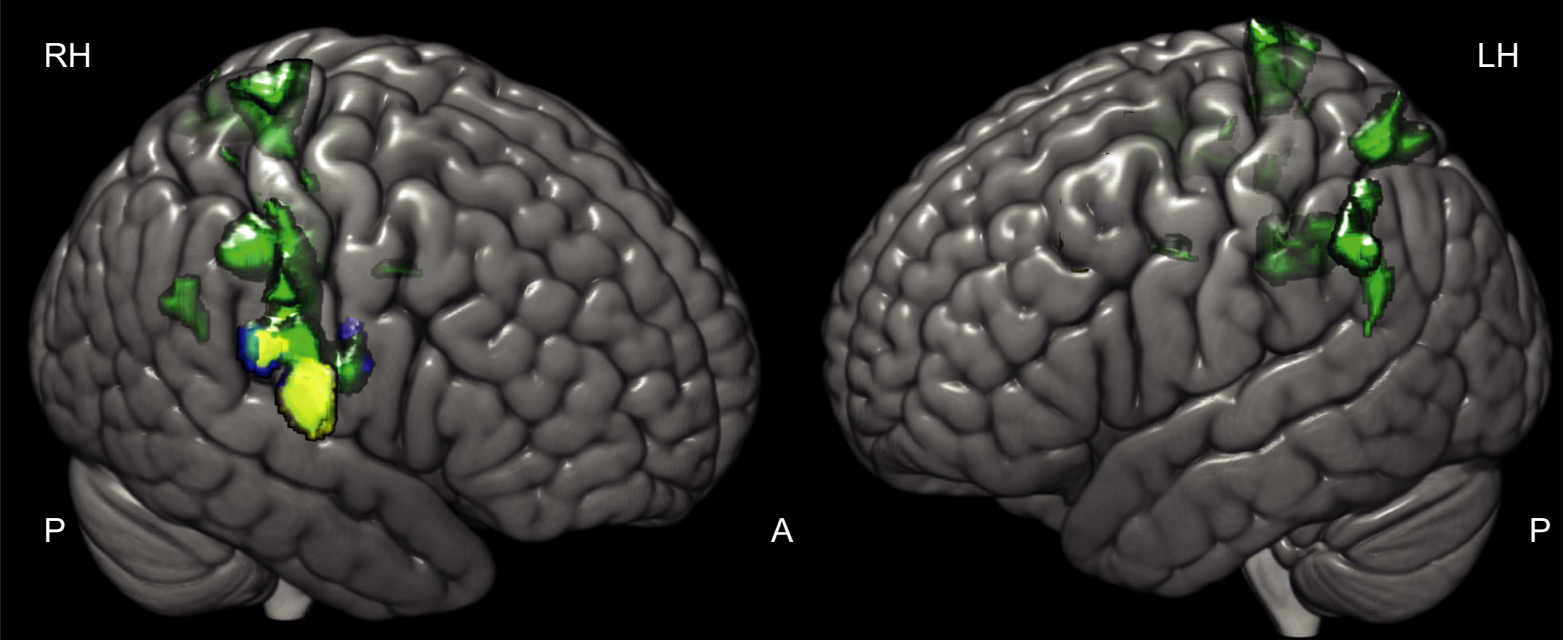
Differing GM reorganizational response size and location in vestibular subtypes of pontomedullary infarcts. Patients with a deviation of the SVV at stroke onset (green) compared to the depiction of the structural follow‐up response in patients with pathological SVV deviation and SPN (yellow), and the response of patients with rotatory vertigo as the initial symptom (blue). Areas associated with the compensation of “pure” vestibular symptoms were located in the right parietal opercular cortex only while areas associated with multisensory integrative function (SVV) showed a bihemispheric distribution along somatosensory cortex and intraparietal sulcus.

For SVV tilts, signal increases were also more pronounced in the right cerebral cortex but involved additional bilateral cortical and subcortical regions. This implies that spontaneous nystagmus and rotatory vertigo represent core vestibular dysfunction, whereas the perception of verticality—an otolith and multisensory achievement—is compensated in the vestibular and somatosensory areas bilaterally with a predominance of some vestibular areas within the right hemisphere.^35^, ^36^, ^37^, ^38^ In other words, the structural changes following infarcts that lead to SVV tilts require the activation of bilateral cortical multisensory areas. (See Summary Figure 6) The findings of the present study further strengthen the theory of right‐sided dominance of vestibular processing and extend it with regard to central compensation of vestibular deficits.

### Volume decreases in visual cortex

Decreases were present in GMV of the visual system (pulvinar, ventral visual stream). A decrease in glucose metabolism and BOLD signal in visual cortex areas has been repeatedly shown in the studies of vestibular stimulation in healthy volunteers and in patients with unilateral peripheral and central vestibular lesions.^17^, ^18^, ^39^ This was attributed to the attempt to minimize a visuo‐vestibular mismatch in visual perception caused by oscillopsia due to nystagmus or by the divergent input in the two sensory systems.^39^ In the current analysis, these changes were also evident on a structural level in the chronic phase, that is, when nystagmus had ended much earlier. It seems that an acute lesion‐induced visual vestibular mismatch and visual vestibular reciprocal interaction cause structural re‐organization of visual and vestibular multisensory brain areas over time.

### Volume decreases in premotor areas and increases in structural multisensory WM connectivity

Additionally, we found decreases in the anterior thalamic nuclei and the premotor cortex in close proximity to but not limited to the FEF (cytoarchitectonic areas 6v, 6d, 6mr, 6mc). A possible explanation could be a profound reduction of voluntary head and neck movements in the acute phase of severe vertigo and spontaneous nystagmus, since these symptoms are aggravated by head movements. While there was a reduction in GMV in these areas, the central vestibular multisensory cortical areas and WM pathways mediating perception of the body in space (superior longitudinal fascicle, SLF) showed a positive response. The SLF provides an anatomical link between the parietal lobe and premotor cortex and is involved in ocular motor coordination, attention, and visuospatial processing, all of which need vestibular and other sensory input to compute maps for spatial orientation.^40^, ^41^ After the infarct‐induced partial loss of vestibular information, a strengthening of these multisensory links is required. This might represent a compensatory perceptual processing strategy for the patients’ disturbance of stance and gait.

### Differences between pontomedullary and pontomesencephalic infarcts

Structural reorganization following graviceptive deficits in pontomedullary infarcts was confined to somatosensory and multisensory cortical areas bilaterally. In contrast, pontomesencephalic infarcts with tilts of the SVV produced far more heterogeneous adaptive changes including frontal, parietal, and middle temporal areas, as well as the striatum. The mean deviation of the SVV between both groups was similar as has been demonstrated before.^42^ The different volumetric changes could be due to the more “integrated” nature of the vestibular pathways in the upper brain stem where the vestibular signals are transformed from a velocity to a position signal.^43^, ^44^, ^45^ This signal is further integrated in the thalamus and cortex where it is needed for spatial orienting and navigation and the modulation of motor output.

With respect to lateralization, an effect of lesion site (R vs L) has to be accounted for, because the majority of pontomesencephalic infarcts was left sided.

### Limitations

We were not able to differentiate between compensatory changes following right‐sided vs left‐sided brain stem infarcts. However, in the case of pontomedullary infarcts, we found a strong right‐hemispheric dominance of volumetric changes where the infarcts were equally distributed between both sides. Therefore, we do not expect significant effects of lesion side in the brain stem on cortical vestibular compensation. Still, a sufficiently powered statistical analysis of this effect would be an interesting topic for further analysis. Furthermore, we were not able to compare our data with infarcts that did not elicit vestibular or ocular motor deficits separately, probably due to the high degree of interconnection of the two systems at the level of the brain stem. Based on our clinical data in which all patients included in the study suffered from some degree of oculomotor or vestibular dysfunction, this problem may even turn out to be impossible to separate in future research, since these pathways are running through the whole brain stem.

Despite the long period of patient recruitment for our study, we were not able to include a substantial enough number of left‐handed patients to warrant a dedicated analysis. The effect of handedness on central vestibular compensatory processes therefore remains unclear.

Further, the use of different scanners presents a bias that is owed to the long recruiting period. However, patients and controls were evenly balanced over the scanners and all patients received their longitudinal MRIs on the same scanner. Furthermore, the raw 3D resolution of the sequences and field strength was identical. We obtained high data quality estimates over the complete sample and good to very good signal homogeneity for the different tissue types in the quality control evaluation as part of the CAT12 toolbox. A side effect to further reduce the role of a scanner‐effect bias is our application of rigorous permutation testing which would have resulted in a null‐finding if the signal quality (noise level) between the scanner types had differed much. Therefore, while the use of different scanners represents a potential limitation of the study, we are confident that it did not bias our results in a negative way.

To correct for the moderate sample sizes in the respective groups we applied rigorous methodological scrutiny in the chosen methods (*TFCE, FWE* correction for multiple comparisons).

Lastly, we had to exclude patients with bilateral or multiple infarcts within the vertebrobasilar territory and those who needed prolonged mechanical ventilation. Therefore, there is an inherent bias to smaller infarcts and lesser clinical symptoms in our patient sample.

## Conclusions

This study, for the first time, demonstrates substantial neuroplasticity in both hemispheres along with the clinical compensation of vestibular deficits following unilateral brain stem infarcts.

For patients with incomplete remission of vestibular symptoms, for instance, noninvasive brain stimulation of the right parietal opercular cortex could be an interesting treatment option to boost cortical compensation.

## Conflict of Interest

There are no conflict of interest.

## Author Contributions

JC involved in acquisition and analysis of data, drafting a significant portion of the manuscript and figures; MH, RB, ME, VK, IS, OE, TS, FW, and MD involved in acquisition and analysis of data; SBB involved in acquisition and analysis of data, study concept and design; PzE involved in analysis of data, drafting a significant portion of the manuscript and figures; MD involved in study concept and design, drafting a significant portion of the manuscript.
